# Identification and validation of prognostic genes related to centrosome amplification in multiple myeloma

**DOI:** 10.7717/peerj.21283

**Published:** 2026-06-04

**Authors:** Pengjun Liao, Jiaqi Tan, Lingji Zeng, Peilong Lai, Xiaojuan Wei

**Affiliations:** 1 Department of Hematology, Guangdong Provincial People’s Hospital (Guangdong Academy of Medical Sciences), Southern Medical University, Guangzhou, China; 2 Department of Lymphoma, Guangdong Provincial People’s Hospital (Guangdong Academy of Medical Sciences), Southern Medical University, Guangzhou, China

**Keywords:** Multiple myeloma, Centrosome amplification, Prognostic genes, Risk model, Immune microenvironment

## Abstract

**Background:**

Prognostic biomarkers associated with centrosome amplification-related genes (CARGs) in multiple myeloma (MM), a malignancy originating from bone marrow plasma cells, remain incompletely defined. This study aimed to identify CARG-associated prognostic genes in MM.

**Methods:**

Transcriptome data for MM and CARGs were obtained from public databases. Initially, differentially expressed genes (DEGs) from the GSE6477 dataset were intersected with CARGs to identify candidate genes, whose biological functions were then analyzed. Regression analyses were conducted to identify prognostic genes linked to CA, from which a risk model for stratification was developed. Further analyses included nomogram construction, functional pathway analysis, immune microenvironment assessment, immunotherapy response evaluation, and drug sensitivity testing. Prognostic gene expression was validated by reverse transcription-quantitative PCR (RT-qPCR).

**Results:**

Forty candidate genes were identified from the intersection of 946 DEGs and 784 CARGs, which were associated with biological processes such as spindle organization. Six prognostic genes—*RUVBL1*, * ARL2*, * KLHL12*, * ECT2*, * SAC3D1*, and * CCND1*—were identified. A risk model and nomogram with superior predictive accuracy were constructed. These genes were found to influence MM pathogenesis by affecting pathways such as translation, pre-mRNA processing, retinoblastoma gene regulation in cancer, mitochondrial translation, and ribosome biogenesis. Additionally, immune cell infiltration, including naive B cells, immunotherapy responses, and drug sensitivities, particularly to CCTOO7093, were impacted by risk scores. RT-qPCR validation confirmed a significant up- regulation of these six genes in MM.

**Conclusion:**

Six prognostic genes associated with CA in MM were identified, and a predictive risk model was developed, offering valuable insights for clinical prognosis and immunotherapy in MM.

## Introduction

Multiple myeloma (MM) is the second most common hematologic malignancy, accounting for approximately 10% of all hematologic cancers. It is characterized by clonal proliferation of plasma cells in the bone marrow, often leading to severe complications such as osteolytic lesions, renal impairment, and immunodeficiency (Fang et al., 2021; Rajkumar, 2024). Over the past two decades, the introduction of novel agents—including proteasome inhibitors (*e.g.*, bortezomib), immunomodulatory drugs (*e.g.*, lenalidomide), anti-CD38 monoclonal antibodies (*e.g.*, daratumumab), and B cell maturation antigen (BCMA)-targeted CAR-T cell therapies—has significantly extended median overall survival, reaching over 10 years in some patients (Garfall, 2024; Soekojo & Chng, 2022). However, due to clonal evolution, acquired drug resistance, and the immunosuppressive tumor microenvironment, most patients eventually relapse. This challenge is particularly pronounced in those harboring high-risk cytogenetic abnormalities (such as del17p or t(4;14)) or those with multi-drug resistant disease, for whom current treatment options remain limited and outcomes are poor (Grosicki, Bednarczyk & Kociszewska, 2023; Soekojo & Chng, 2022). Therefore, there is an urgent need to identify novel prognostic biomarkers to refine risk stratification, guide personalized treatment, and explore potential therapeutic targets in MM.

Centrosome amplification (CA), defined as an abnormal increase in centrosome number (≥3 per cell), disrupts mitotic spindle assembly and drives chromosomal instability (CIN) and aneuploidy, thereby serving as a key driver of tumor initiation and progression (Favasuli et al., 2024). In MM, CA is frequently observed and is closely associated with early disease development, hyperdiploid karyotypes, and unfavorable prognosis (Dementyeva et al., 2013; Kryukova, Kryukov & Hajek, 2016). Mechanistic studies have revealed that common genetic alterations in MM, such as DIS3 deletion or 1q21 amplification, can induce CA by interfering with RNA metabolism or DNA damage repair pathways, further exacerbating genomic instability (Favasuli et al., 2024; Marchesini et al., 2017). Although the clinical relevance of CA in MM is increasingly recognized, a systematic screening of CA-related genes (CARGs) and a comprehensive evaluation of their prognostic value remain lacking, hindering the translation of CA-associated biomarkers into clinical practice.

To address this knowledge gap, we integrated MM transcriptomic data from the Gene Expression Omnibus (GEO) database with CARGs derived from the Gene Ontology (GO) database, employing bioinformatics approaches to systematically identify CARGs associated with prognosis in MM. We identified and validated a CARG-based signature as a novel prognostic biomarker for MM. Through differential expression analysis, functional enrichment analysis, and machine learning-based modeling, we constructed a robust risk stratification tool, evaluated its associations with the tumor immune microenvironment and therapeutic response, and assessed its clinical potential in guiding personalized treatment strategies. This study aims to provide new insights into the field of precision medicine in MM, with a focus on clinical application rather than detailed mechanistic exploration of individual genes in MM biology.

## Materials & Methods

### Data source

The overall workflow of the study is shown in Fig. S1. First, the research data were obtained from three publicly available array sequencing datasets in the GEO database. The GSE6477 dataset (platform: GPL96) served as the primary dataset for differential gene expression analysis, which included 75 bone marrow plasma cell samples from patients with MM and 15 samples from healthy donors. For survival analysis and model construction, two independent cohorts with complete OS information were used: the GSE136337 dataset (GPL27143) to construct a risk model with 426 MM bone marrow samples, and the GSE24080 dataset (GPL570) to validate the risk model with 559 MM bone marrow samples. A total of 784 CARGs were obtained from the GO database under the term GO:0061524 (CA).

It should be noted that none of the above three GEO datasets provided direct information on CA status. Therefore, the analysis in this study focused on the expression characteristics of CARGs rather than directly determining the CA status of the samples.

### Functional pathways and protein-protein interaction (PPI) analyses of candidate genes

First, differential expression analysis was performed on MM and normal samples in the GSE6477 dataset using the limma package (v3.54.1) (Ritchie et al., 2015), with criteria set at —log_2_ fold change (FC)—>1 and adjusted *P* < 0.05. Visualization was conducted with the ggplot2 (v3.5.0) (Gustavsson et al., 2022) and pheatmap (v1.0.12) (Gu & Hubschmann, 2022) packages. Candidate genes were identified by intersecting differentially expressed genes (DEGs) and CARGs *via* the VennDiagram package (v1.7.1) (Chen & Boutros, 2011).

Subsequently, GO analysis was conducted using the clusterProfiler package (v4.2.2) (Wu et al., 2021) to explore the biological functions of candidate genes, with the criterion set at *P* < 0.05. For PPI network analysis, the STRING database (https://cn.string-db.org/) was used to construct the network with a confidence score threshold of >0.4, and the interacting proteins were visualized using Cytoscape software (v3.1.1) (Shannon et al., 2003).

### Identification of prognostic genes

To identify OS-related genes in patients with MM, candidate genes in the GSE136337 dataset were first analyzed using the survival package (v3.5-3) (Lei et al., 2023), including univariate Cox analysis (hazard ratio [HR] ≠ 1, *P* < 0.05) and the proportional hazard (PH) assumption test (*P* > 0.05). Results from univariate Cox analysis were visualized with the forestplot package (v2.0.1) (Li, Lu & Yin, 2022). Prognostic genes were then identified from the remaining candidate genes *via* least absolute shrinkage and selection operator (LASSO) regression analysis using the glmnet package (v4.1-4) (Li, Lu & Yin, 2022).

### Development and validation of a risk model

Based on the prognostic genes identified in the GSE136337 dataset, a risk model was developed following this formula (where coef and expr represent the LASSO regression coefficient and gene expression, respectively):

\begin{eqnarray*}\text{Risk score}=\sum _{\mathrm{i}=1}^{\mathrm{n}}\mathrm{coef}({\mathrm{gene}}_{\mathrm{ i}})\times \mathrm{expr}({\mathrm{gene}}_{\mathrm{i}}). \end{eqnarray*}

The optimal risk score cut-off values were determined using the surv_cutpoint function. Patients with survival data were categorized into the high-risk group (HRG) and the low-risk group (LRG). Risk score curves, survival status distribution plots, and heatmaps of prognostic gene expression were generated using the ggplot2 package (v3.5.0). Kaplan–Meier (KM) survival curves were plotted for patients with complete survival information using the survminer package (v0.4.9) (log-rank test, *P* < 0.05). Receiver operating characteristic (ROC) curves for 3-year, 5-year, and 7-year OS were generated using the survivalROC package (v1.0.3) (Heagerty, Lumley & Pepe, 2000), and model effectiveness was evaluated based on the area under the curve (AUC > 0.6) for each time interval (Meng et al., 2021; Xing et al., 2021). The use of AUC at multiple time points in ROC curve analysis mitigates the limitations of evaluating AUC at a single time point, enabling a comprehensive and dynamic assessment of the predictive performance of the risk stratification model over various clinical follow-up periods. To further evaluate the accuracy and generalizability of the risk model, it was validated in the GSE24080 dataset. Additionally, the AUC, accuracy, sensitivity, specificity, and F1 score of the risk model in the GSE136337 dataset were calculated using the caret package (v6.0-94) (https://CRAN.R-project.org/package=caret).

### Independent prognostic analysis and nomogram establishment

In the GSE136337 dataset, the Wilcoxon rank sum test (*P* < 0.05) was used to analyze the distribution differences of risk scores across subgroups stratified by age, gender, International Staging System (ISS) stage, Revised ISS (RISS) stage, race, albumin, β2-microglobulin (B2M), lactate dehydrogenase (LDH), and the percentage of plasma cells in bone marrow (Pc_combest). Survival analysis was conducted using the survminer package (v0.4.9) to examine survival differences between HRG and LRG groups across different clinical features (*P* < 0.05).

To identify independent prognostic factors, clinical variables were sequentially analyzed using univariate Cox analysis (HR ≠ 1, *P* < 0.05), the PH assumption test (*P* > 0.05), and multivariate Cox analysis (HR ≠ 1, *P* < 0.05), with the remaining variables defined as independent prognostic factors. The multicollinearity of independent prognostic factors was assessed using the variance inflation factor (VIF) method. A VIF value >10 indicated severe multicollinearity, a value between five and 10 indicated moderate multicollinearity, and a value <5 indicated low multicollinearity.

Using the identified independent prognostic factors, a nomogram was developed using the rms package (v6.7-0) (Sachs, 2017) to predict the survival probabilities of patients with MM at 3, 5, and 7 years. Calibration and ROC curves were then used to evaluate the calibration and discrimination abilities of the model. The rms package (v6.7-0) and timeROC package (v0.4) (Blanche, Dartigues & Jacqmin-Gadda, 2013) were used to plot the calibration and ROC curves, respectively.

### Gene set enrichment analysis (GSEA)

GSEA was performed to investigate the pathways and biological mechanisms associated with prognostic genes. The reference gene set “c2.cp.v2023.2.Hs.symbols.gmt” was obtained *via* the msigdbr package (v7.5.1) (Liberzon et al., 2015). In the GSE136337 dataset, samples were categorized into high- and low-expression groups based on prognostic gene expression, with genes ranked by log_2_FC values in descending order. GSEA was performed for each prognostic gene using the clusterProfiler package (v4.2.2), with thresholds set at false discovery rate (FDR) < 0.05 and *P* < 0.05. The top five most significant pathways for each prognostic gene were visualized using the enrichplot package (v1.18.3) (Wang et al., 2022).

### Immune microenvironment, immunotherapy response, and drug sensitivity analyses

In the GSE136337 dataset, the immune microenvironment of HRG and LRG was assessed. The relative proportions of 28 immune infiltrating cell types (PMID: 36845727) were evaluated using the single-sample GSEA (ssGSEA) algorithm. Differential immune infiltrating cell types between the two groups were identified using the Wilcoxon rank sum test (*P* < 0.05). Spearman correlation analysis was performed with the psych package (v2.2.9) (Orifjon et al., 2023) to assess correlations among differential immune infiltrating cell types, as well as between these cell types and prognostic genes, with thresholds set at —correlation (cor)— > 0.3 and *P* < 0.05.

In the GSE136337 dataset, tumor immune dysfunction and exclusion (TIDE) scores for HRG and LRG were calculated using the TIDE official website (http://tide.dfci.harvard.edu/) to evaluate intergroup differences in immunotherapy response. The Wilcoxon rank sum test (*P* < 0.05) was used to assess TIDE score differences between groups. Spearman correlation analysis (—cor— > 0.3, *P* < 0.05) was performed with the psych package (v2.2.9) to explore the association between TIDE score and risk score.

Finally, in the GSE136337 dataset, based on 138 chemotherapy drugs provided by the Genomics of Drug Sensitivity in Cancer (GDSC) database (https://www.cancerrxgene.org/), the 50% inhibitory concentration (IC_50_) values for MM samples in risk groups were computed using the pRRophetic package (v0.5) (Geeleher, Cox & Huang, 2014). The IC_50_ differences for the drugs were assessed using the Wilcoxon rank sum test (*P* < 0.05).

### Association analysis with centrosome amplification-related features

To evaluate the association between the risk model and biological features related to centrosome amplification (CA), surrogate markers associated with CA were constructed. First, based on the common 1q21 amplification region in multiple myeloma (Hanamura, 2021), representative genes including *CKS1B*, *PSMD4*, and *BCL9* (Burroughs Garcia et al., 2021; Iriyama et al., 2018) were selected. The average expression level of these genes in each sample was used as the CA score to characterize molecular features associated with 1q21 amplification. Subsequently, in the training cohort (GSE136337) and the validation cohort (GSE24080), patients were divided into high-risk and low-risk groups based on the median risk score. The Wilcoxon rank-sum test was used to compare CA scores between the two groups, and Spearman correlation analysis was performed to evaluate the correlation between the risk score and CA score. A two-sided *P* < 0.05 was considered statistically significant.

### Reverse transcription-quantitative polymerase chain reaction

For gene expression validation, peripheral blood samples were collected from five newly diagnosed patients with MM (meeting International Myeloma Working Group diagnostic criteria, with no prior systemic therapy) and five age-matched healthy controls. Total RNA was extracted from 600 µl of whole blood using TRIzol reagent (chloroform-isopropanol purification), with RNA quality assessed by electrophoresis and purity confirmed using a NanoPhotometer (A260/A280 ∼1.8–2.0). First-strand cDNA was synthesized from 2 µg total RNA using the Hifair^®^ III reverse transcription kit (Yeasen), and quantitative polymerase chain reaction (qPCR) was performed on a BIO-RAD CFX Connect system with SYBR Green Master Mix (triplicate reactions: 95 °C for 1 min, followed by 40 cycles of 95 °C for 20 s, 55 °C for 20 s, and 72 °C for 30 s). Primer sequences are listed in Table 1. Relative expression levels were calculated using the 2^−^^ΔΔCt^ method (GAPDH as the endogenous control), and statistical significance was determined by an unpaired *t*-test (*P* < 0.05).

This study adhered to the Declaration of Helsinki and was approved by the Ethics Committee of Guangdong Provincial People’s Hospital (KY2025-651-01), with written informed consent obtained from all participants.

### Statistical analysis

Bioinformatics analyses were performed using R language (v4.2.2) (Staples, 2023). The Wilcoxon rank sum test was used to assess group differences, with *P* < 0.05 considered significant.

**Table 1:** Primers for prognostic genes and the internal reference gene (GAPDH).

**Primer**	**Sequence (5′ to 3′)**
RUVBL1-F	TGGAGTCTTCTATCGCTCCCA
RUVBL1-R	CAGCTGCACTGAGTACCTGTTT
ARL2-F	GGTGGAGGAGGTCCTGGAG
ARL2-R	GTGAAGGTTGAGGGACCTGG
KLHL12-F	GCCTGGGTATTAGGGATTTTGC
KLHL12-R	CGCACTTGATTAGCTTTTCCAC
ECT2-F	TGGCTCTTAGAGAGAGCAGC
ECT2-R	AAGCCCTAGGAGTTCCACCT
SAC3D1-F	AGGAGCCCACCATGACAGT
SAC3D1-R	TTGACCATGAAGCCCAGAGG
CCND1-F	GCTGCGAAGTGGAAACCATC
CCND1-R	CCTCCTTCTGCACACATTTGAA
GAPDH-F	ATGGGCAGCCGTTAGGAAAG
GAPDH-R	AGGAAAAGCATCACCCGGAG

**Notes.**

GAPDH Glyceraldehyde-3-Phosphate Dehydrogenase

## Results

### Related functional pathways and complex PPI network of candidate genes

**Figure 1:**
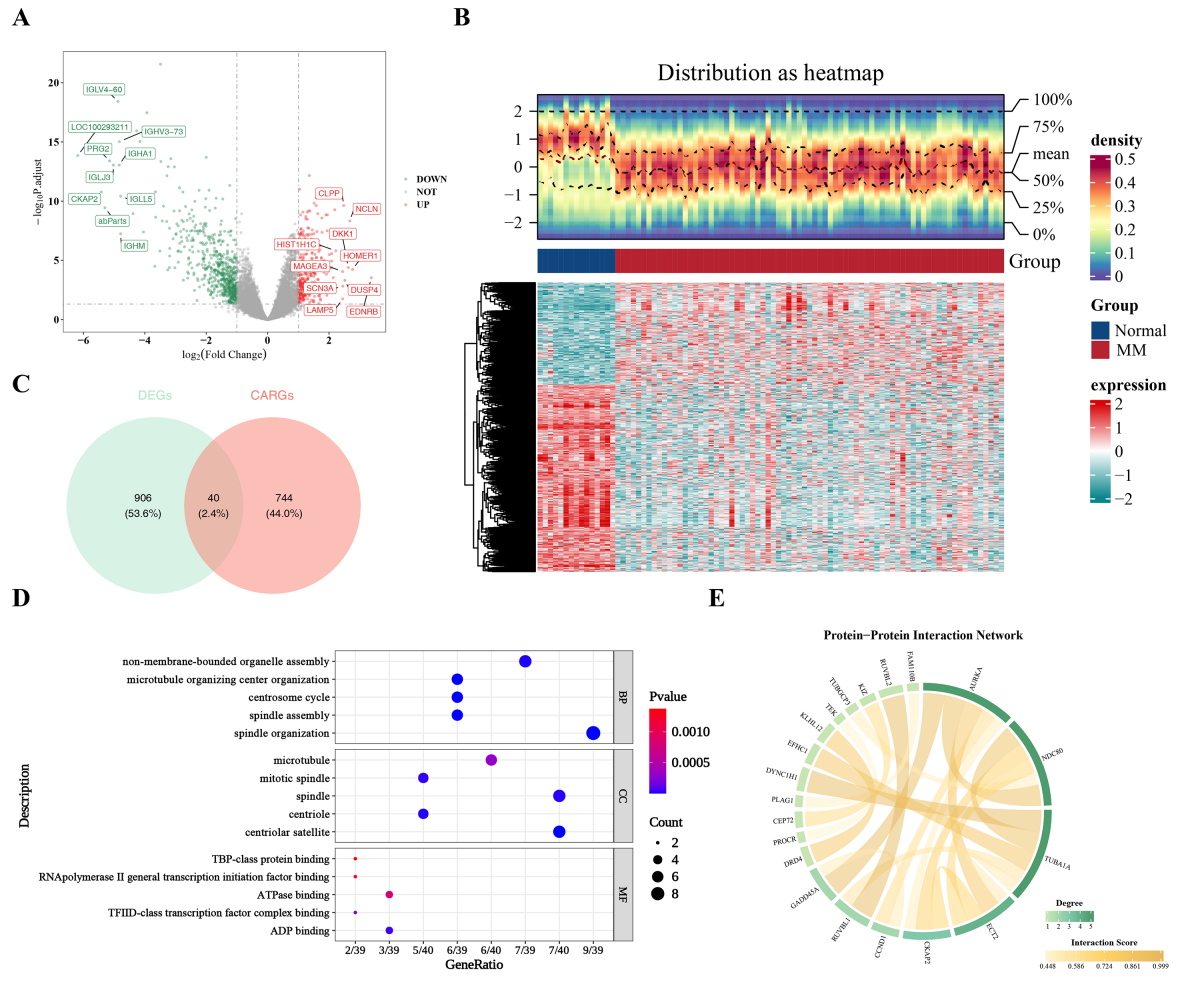
Function and PPI network of candidate genes. (A) Volcano plot and (B) jheatmap of DEGs. (C) Venn diagram of the intersection between DEGs and key CARGs. (D) GO and KEGG enrichment analysis of candidate genes. (E) PPI network of candidate genes.

In the GSE6477 dataset, 946 DEGs were identified between MM and normal groups, including 334 upregulated and 612 downregulated genes (Figs. 1A–1B). Subsequent intersection of these DEGs with key CARGs revealed 40 candidate genes in MM (Fig. 1C, Table S1).

These 40 candidate genes were significantly enriched in 112 GO terms, categorized into 74 biological processes (BP), 32 cellular components (CC), and six molecular functions (MF). The top five most significant entries per category highlighted key functional associations with spindle organization (BP), centriolar satellite (CC), and ADP binding (MF) (Fig. 1D), processes closely associated with CA biology.

The PPI network of the 40 candidate genes included 20 interacting proteins and 20 PPI pairs (Fig. 1E), reflecting the complex interactions among CA-related proteins and their potential relevance to MM.

### Identification of six prognostic genes

**Figure 2:**
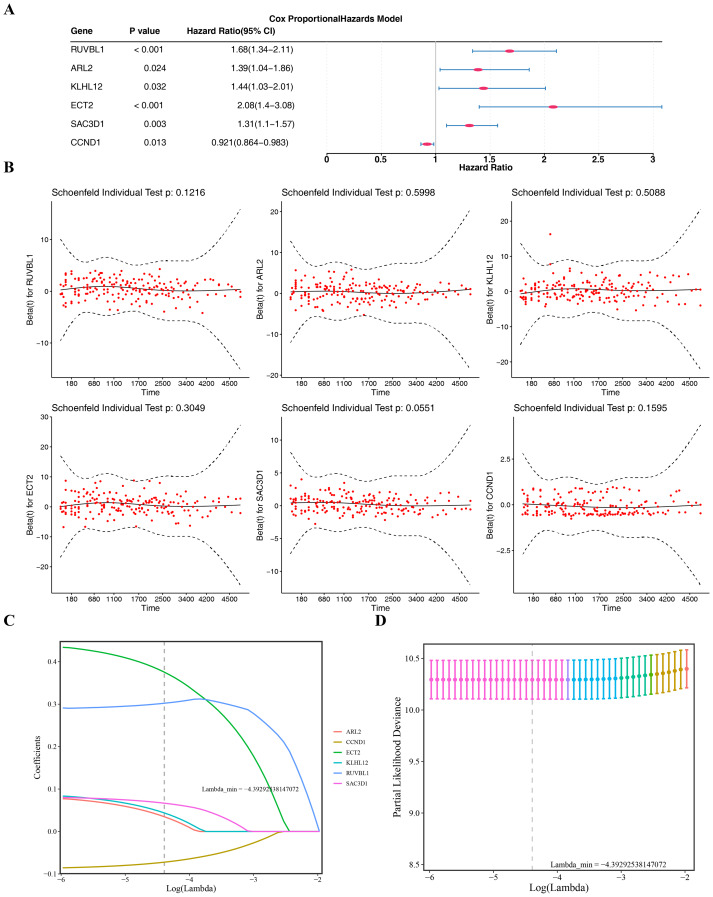
Identification of prognostic genes. (A–B) Forest plot and PH assumption test of univariate Cox regression. (C–D) LASSO regression coefficient path plot and cross-validation curve.

In the GSE136337 dataset, univariate Cox analysis (*P* < 0.05) and the PH assumption test (*P* > 0.05) identified six CA-associated candidate prognostic genes (Figs. 2A–2B). Among these, *CCND1* was associated with favorable prognosis (HR < 1), while *RUVBL1*, *ARL2*, *KLHL12*, *ECT2*, and *SAC3D1* were linked to poor prognosis (HR > 1). LASSO regression analysis confirmed these six genes as prognostic markers (lambda min = −4.39292538147072) (Figs. 2C–2D).

### Development of a risk model with moderate predictive ability

A risk model was constructed based on the six prognostic genes using the formula:

risk score = (0.3022) ×*RUVBL1* expression + (0.0349) × *ARL2* expression + (0.0439) × *KLHL12* expression + (0.3756) × *ECT2* expression + (0.0668) × *SAC3D1* expression + (−0.0723) × *CCND1* expression.

**Figure 3:**
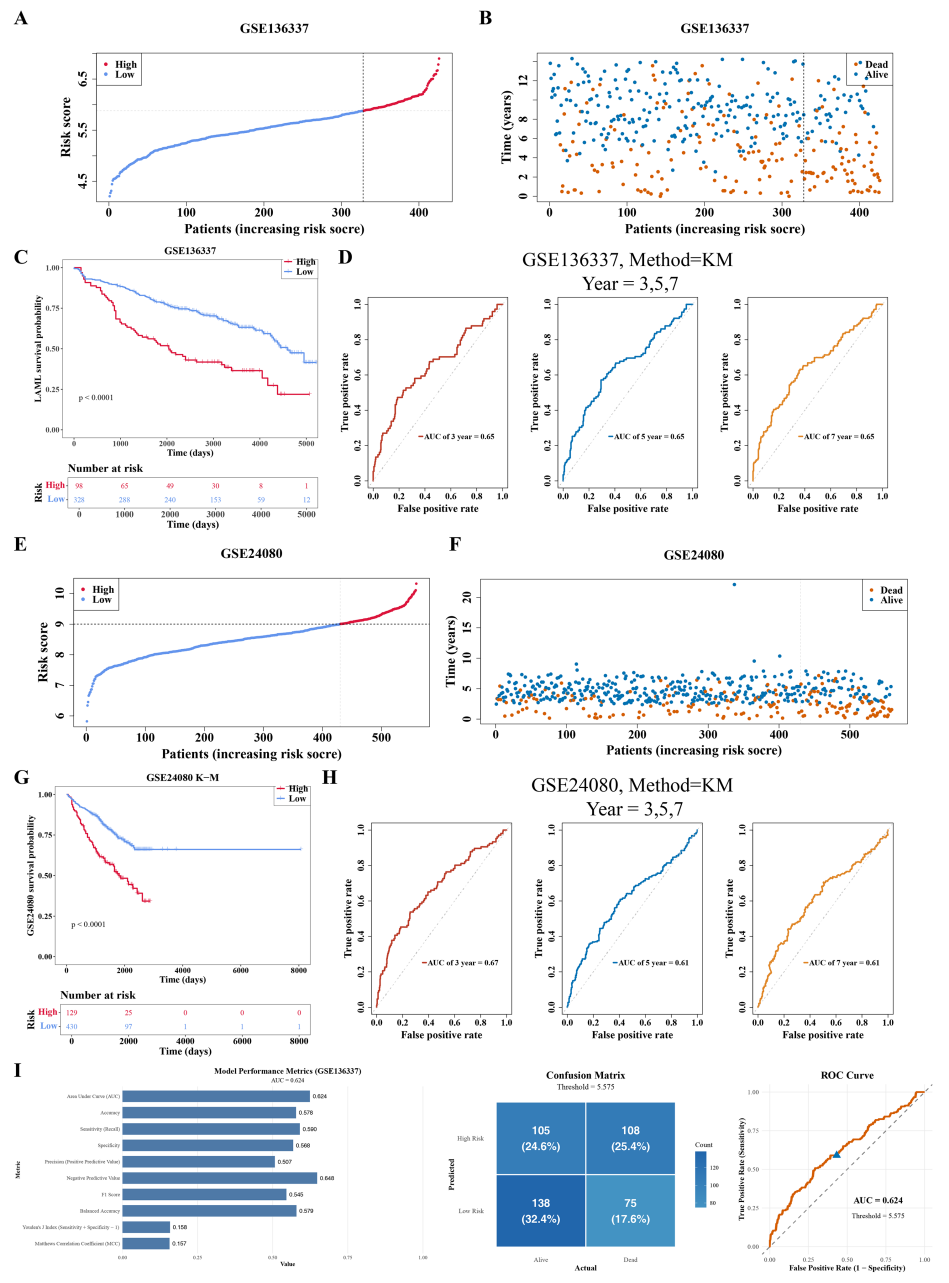
Development and verification of risk model. (A–B) Risk score curves and survival outcome distributions of the GSE136337 dataset. (C–D) K-M survival curves and ROC curves of the GSE136337 dataset. (E–F) Risk score curves and survival outcome distributions of the GSE24080 dataset. (G–H) K-M survival curves and ROC curves of the GSE24080 dataset. (I) Bar plot of risk model performance metrics (AUC value, accuracy, sensitivity, specificity, and F1 score), confusion matrix of the risk model, and ROC curve for evaluating the discriminative ability of the model.

The 426 MM samples in the GSE136337 dataset were stratified into high-risk (HRG, 98 samples) and low-risk (LRG, 328 samples) groups based on the optimal cutoff value of the risk score (5.880471). Risk score and survival status distributions showed an increase in mortality among MM individuals with higher risk scores (Figs. 3A–3B). KM survival analysis indicated significantly higher survival probabilities in LRG (*P* < 0.0001) (Fig. 3C), and the model demonstrated AUC values >0.6 for 3-, 5-, and 7-year survival prediction, confirming its prognostic predictive efficacy (Fig. 3D).

The CA-associated risk model was further validated in the GSE24080 dataset. A total of 559 MM samples were stratified into HRG (129 samples) and LRG (430 samples) groups based on the optimal cutoff value of the risk score (9.004242). Risk score and survival status distributions (Figs. 3E–3F), KM survival curves (*P* < 0.0001) (Fig. 3G), and ROC curves (AUC > 0.6) (Fig. 3H) showed high consistency with the results from the GSE136337 dataset. Additionally, the AUC value for the GSE136337 dataset was calculated at 0.624, indicating the model’s moderate discriminative ability. Sensitivity (0.590) and specificity (0.568) were relatively close, demonstrating the model’s balanced ability to identify samples in both groups. However, accuracy was only 57.8%, just 7.8 percentage points higher than random guessing (50%), and the F1 score was relatively low at 54.5% (Fig. 3I). In summary, the risk model demonstrated basic discriminative capacity but requires further optimization for clinical application.

### Screening of three independent prognostic factors and establishment of a nomogram with outstanding predictive ability

**Figure 4:**
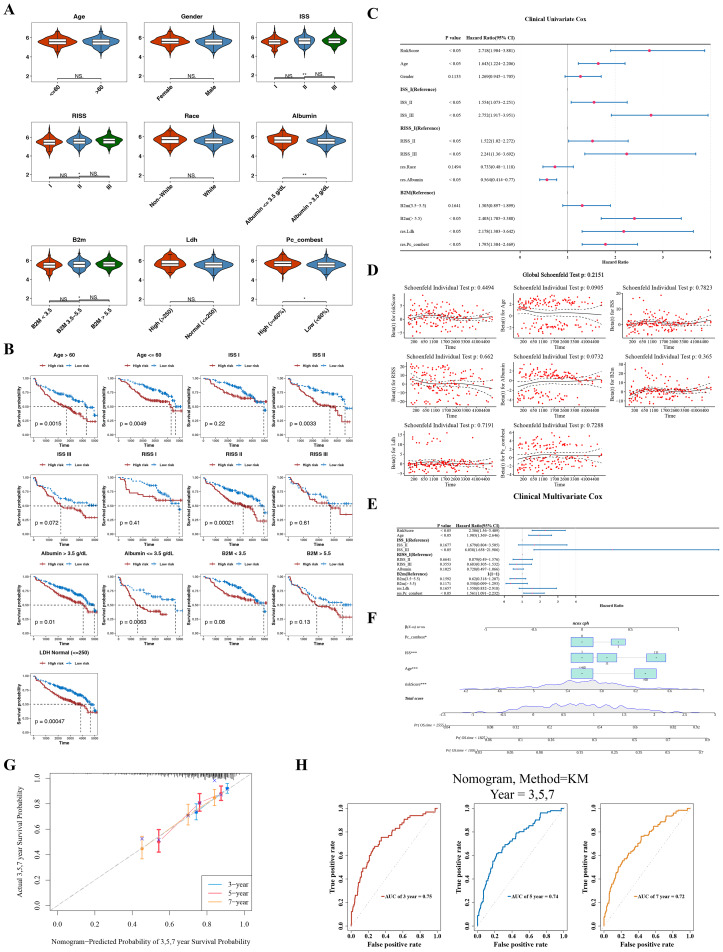
Independent prognostic analysis and establishment and validation of the nomogram model. (A) Differential analysis of risk scores among different clinical characteristic subgroups. (B) Survival differences among subgroups with distinct clinical characteristics. (C–D) Forest plot and PH assumption test of univariate Cox regression. (E) Forest plot of multivariate Cox regression. (F) Nomogram model. (G–H) Calibration curve and receiver operating characteristic (ROC) curve of the nomogram model.

In the GSE136337 dataset, CA-associated risk scores varied significantly across subgroups based on ISS stage I/III, RISS stage I/III, albumin (≤ 3.5 and >3.5 g/dL), B2M (<3.5 and >5.5), and Pc_combest (high (≥ 60%) and low (<60%)) (*P* < 0.05), suggesting correlations between MM staging, albumin, B2M, Pc_combest, and risk scores (Fig. 4A). Additionally, among all clinical features, the survival rate of patients with MM in HRG was significantly lower than that of patients in LRG (Fig. 4B).

Univariate Cox regression analysis revealed that risk score, age, ISS, RISS, albumin, B2M, LDH, and Pc_combest were significantly associated with the prognosis of patients with MM (*P* < 0.05) (Fig. 4C). The PH assumption test confirmed that these variables satisfied the PH assumption for the Cox regression model (*P* > 0.05), and therefore, they were retained for multivariate Cox regression analysis (Fig. 4D). Multivariate Cox analysis (*P* < 0.05) further identified risk score, age, ISS, and Pc_combest as independent prognostic factors (Fig. 4E). VIF analysis showed that the VIF values for risk score, age, ISS, and RISS were all <5 (Table S2), indicating low multicollinearity among these independent prognostic factors.

A nomogram was constructed based on these independent prognostic factors (Fig. 4F). The nomogram demonstrated that higher total points were associated with a higher survival probability. Its calibration curve slope was close to 1, and the AUC values for 3-, 5-, and 7-year survival predictions exceeded 0.7, confirming the model’s excellent predictive accuracy for clinical MM management (Figs. 4G–4H).

### Crucial functional pathways of prognostic genes

**Figure 5:**
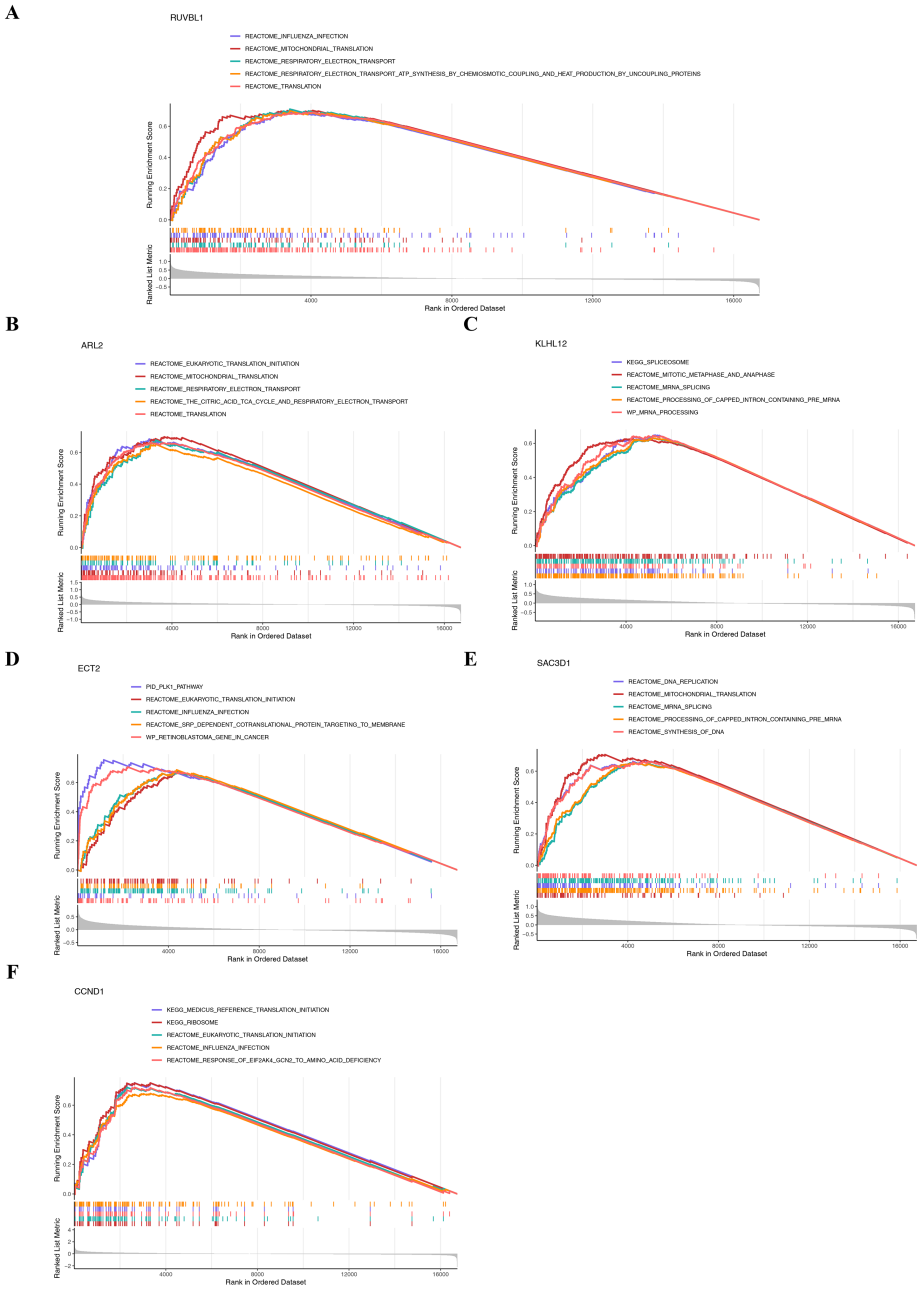
Functional enrichment analysis. (A–F) GSEA analysis of six prognostic genes.

GSEA was performed to explore pathways associated with the six CA-associated prognostic genes. *RUVBL1* and *ARL2* were enriched in 591 and 235 pathways, respectively, with translation as the key representative pathway (Figs. 5A–5B). *KLHL12* was enriched in 1,028 pathways, dominated by the processing of capped intron-containing pre-mRNA pathway (Fig. 5C). *ECT2* was enriched in 835 pathways, with the retinoblastoma (RB) gene in cancer pathway as the main representative (Fig. 5D). *SAC3D1* was enriched in 625 pathways, with mitochondrial translation as the core pathway (Fig. 5E). *CCND1* was enriched in 229 pathways, predominantly involving the ribosome pathway (Fig. 5F). These results consistently highlighted the functional enrichment of each prognostic gene in its respective key pathway.

### Differential immune microenvironment, TIDE scores, and drug sensitivities

**Figure 6:**
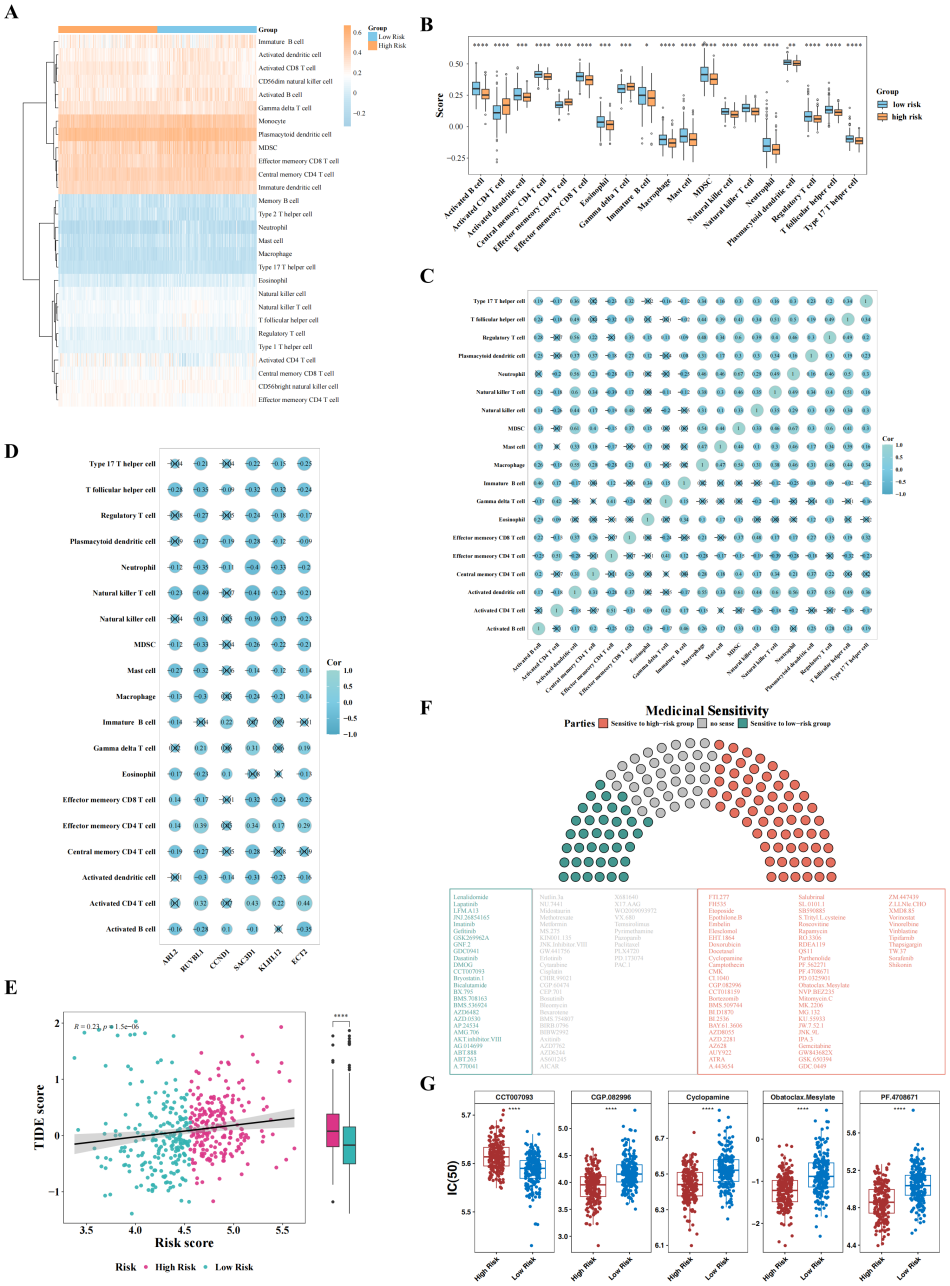
Immune cells, TIDE score, and drug of risk model. (A) Heat map of immune cell infiltration in HR G group and LR G group. (B) Differential analysis of immune cell infiltration between HR G and LRG. (C) Correlation analysis of differential immune cells. (D) Correlation analysis of prognostic genes and differential immune cells. (E) Differential analysis of TIDE score between HR G and LR G. (F–G) Heatmap and boxplot for analysis of drug sensitivity differences between the HRG and LRG.

In the GSE136337 dataset, the infiltration patterns of 28 immune cell types were compared between the CA-associated HRG and LRG (Fig. 6A), with 19 cell types showing significant intergroup differences, including activated B cells, activated CD4 T cells, and activated dendritic cells (Fig. 6B).

Correlation analysis revealed significant associations among differential immune cell types, as well as between these immune cell types and the six CA-associated prognostic genes (Figs. 6C–6D). Notably, a strong positive correlation was observed between neutrophils and myeloid-derived suppressor cells (MDSC) (cor = 0.67, *P* < 0.05), while a significant negative correlation was found between natural killer T cells and effector memory CD4 T cells (cor = −0.39, *P* < 0.05). Additionally, natural killer T cells were most strongly inversely correlated with *RUVBL1* (cor = −0.49, *P* < 0.05), whereas activated CD4 T cells exhibited the strongest positive correlation with *ECT2* (cor = 0.44, *P* < 0.05).

HRG had a significantly higher TIDE score than LRG (Fig. 6E), and a weak yet significant positive correlation was observed between the TIDE score and risk score (cor = 0.23, *P* < 0.05). Drug sensitivity analysis identified 101 drugs with significant intergroup differences in IC_50_ (Fig. 6F). The top five drugs with the most pronounced IC_50_ differences were CCT007093, CGP-082996, cyclopamine, obatoclax mesylate, and PF-4708671 (Fig. 6G). Notably, CCT007093 showed a lower IC_50_ in LRG, while the other four drugs exhibited lower IC_50_ values in HRG.

### The risk model showed a significant association with centrosome amplification-related features

To explore the correlation between the constructed risk model and the molecular characteristics of CA, this study first established a CA score based on representative genes from the 1q21 region. In the training cohort (GSE136337), the CA score of the high-risk group was significantly higher than that of the low-risk group (*P* < 0.001), and the risk score showed a significant positive correlation with the CA score (Spearman *r* = 0.33, *P* < 0.001). In the validation cohort (GSE24080), this result remained consistent, with the CA score in the high-risk group also significantly elevated (*P* < 0.001), and the risk score exhibited a significant positive correlation with the CA score (*r* = 0.28, *P* < 0.001). Further analysis of the representative genes in the 1q21 region indicated that, in the training cohort, CKS1B, PSMD4, and BCL9 were all significantly upregulated in the high-risk group (all *P* < 0.01); in the validation cohort, except for BCL9 (*P* = 0.66), the other genes also showed significant increases in the high-risk group (all *P* < 0.01). These results indicated that the risk model demonstrated consistent changes in both overall scoring and individual gene levels in relation to the 1q21 associated characteristics (Fig. S2).

### RT-qPCR validation of prognostic genes

**Figure 7:**
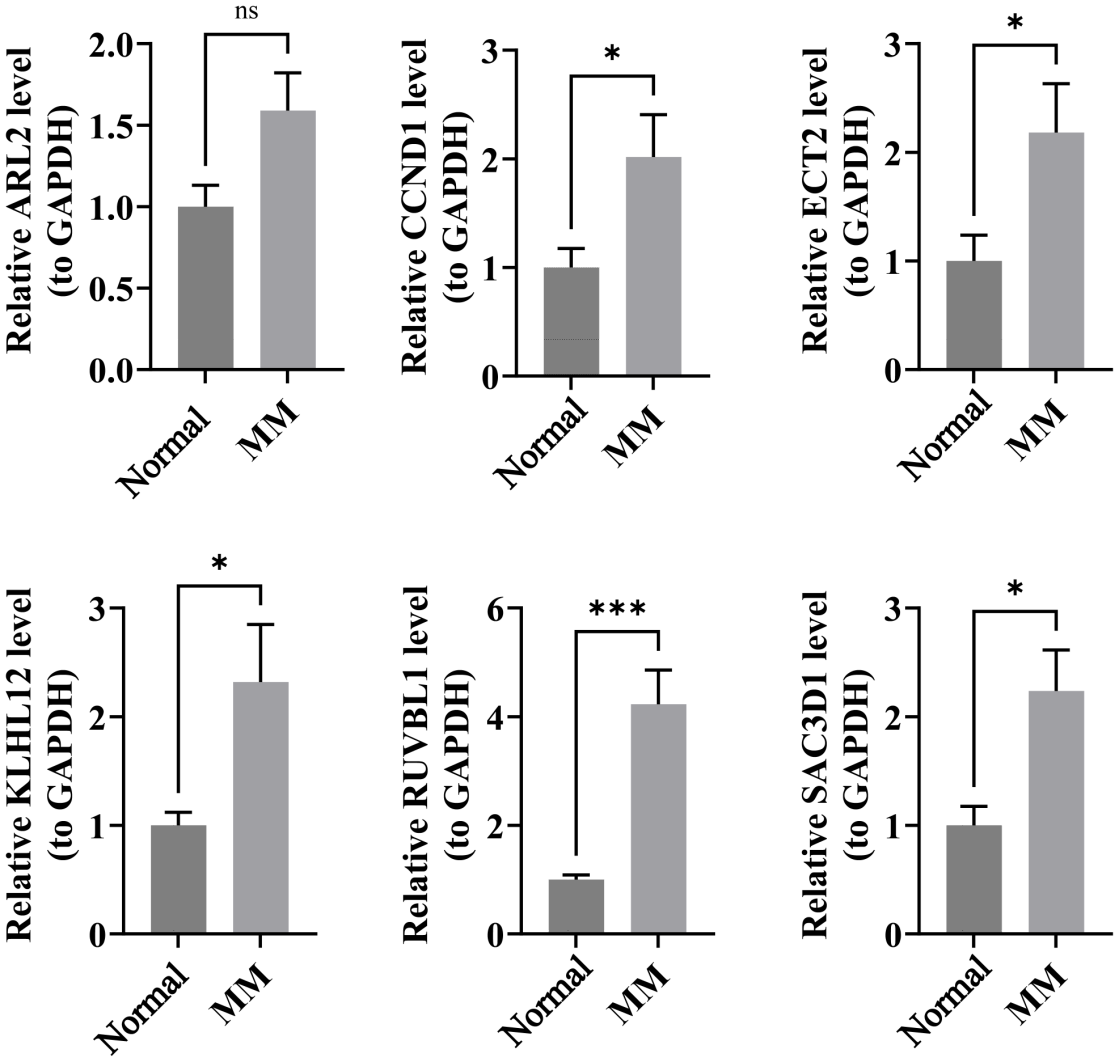
RT-qPCR analysis of prognostic gene expression levels between MM and normal samples. Compared to the normal group, the six CA-associated prognostic genes exhibited distinct expression patterns in MM samples: RUVBL1, KLHL12, ECT2, SAC3D1, and CCND1 were significantly upregulated (*P* < 0.05), while ARL2 showed an upward trend without statistical significance.

Compared to the normal group, the six CA-associated prognostic genes exhibited distinct expression patterns in MM samples: *RUVBL1*, *KLHL12*, *ECT2*, *SAC3D1*, and *CCND1* were significantly upregulated (*P* < 0.05), while *ARL2* showed an upward trend without statistical significance (Fig. 7).

## Discussion

MM, a hematological malignancy characterized by clonal plasma cell dysplasia, is marked by significant genomic instability often driven by CA. As a hallmark of mitotic dysregulation, CA induces chromosome segregation errors and clonal evolution, fostering genomic chaos even in MM, which typically has a low proliferative capacity (Dementyeva et al., 2013; Kryukova, Kryukov & Hajek, 2016). In the present study, a six-gene CARG signature (*RUVBL1*, *ARL2*, *KLHL12*, *ECT2*, *SAC3D1*, *CCND1*) was identified, enabling robust risk stratification in MM. The risk model developed based on this signature showed consistent prognostic performance across independent cohorts and was correlated with perturbations in the tumor immune microenvironment and differential drug sensitivities. Mechanistically, this signature converges on pathways essential for mitotic regulation and genomic stability—core processes driving CA. While previous studies have linked CARGs to centrosome homeostasis and mitotic spindle assembly, whose dysregulation accelerates MM progression, our findings extend this knowledge by demonstrating that a composite CARG signature, rather than individual genes, effectively captures CA-associated prognostic heterogeneity. This highlights the potential of the signature as a clinically actionable tool for MM risk assessment and therapeutic stratification.

This study identified a six-gene CARG signature (*RUVBL1*, *ECT2*, *CCND1*, *ARL2*, *KLHL12*, *SAC3D1*) that acts synergistically to drive MM progression. Mechanistically, the signature centers on mitotic regulation, RNA metabolism, and metabolic adaptation—key pathways linked to CA and genomic instability in MM. *ECT2* (critical for mitotic entry) and *CCND1* (key for G1/S transition) directly connect the signature to CA-driven CIN, consistent with their known roles in MM cell cycle dysregulation (He et al., 2024; Yamaguchi et al., 2009). *CCND1* is upregulated in multiple myeloma but acts as a protective factor in the risk model (HR < 1). This apparent paradox may reflect a compensatory response: given the disease’s hallmark G1/S checkpoint dysregulation and CDK hyperactivation (Iriyama et al., 2018), *CCND1* upregulation could partially restore cell-cycle control and mitigate damage from excessive proliferation pressure (Wang et al., 2020). Yet this compensation is insufficient to overcome overall malignant progression—hence its protective weight in the model despite elevated expression. This duality illustrates how oncogenic drivers and adaptive responses coexist in tumor biology. *RUVBL1*, *KLHL12*, and *SAC3D1* converge on RNA processing and translational control, supporting malignant protein synthesis; for instance, *RUVBL1* enhances MYC-driven transcription and ribosome biogenesis, a well-established oncogenic axis in MM (Assimon et al., 2019). *ARL2* links the signature to mitochondrial function and microtubule dynamics, potentially aiding metabolic adaptation to stress and promoting CA-associated tumor cell survival. These findings indicate that the six-gene signature captures the coordinated dysregulation of CA-relevant pathways, rather than the effects of individual genes. This integrated approach aligns with established mechanisms of genomic instability in MM, highlighting the signature’s utility as a proxy for CA-associated prognostic heterogeneity.

The six-gene CARG signature identified in this study converges on five core pathways (translation, capped intron-containing pre-mRNA processing, RB signaling, mitochondrial translation, ribosome biogenesis), which collectively mediate CA-driven genomic instability and metabolic reprogramming in MM. These pathways orchestrate adaptations that are critical for MM progression and therapeutic resistance (Adamia et al., 2020; Cortes et al., 2023).

Mechanistically, the CARG signature is closely linked to MM oncogenic pathways: *CCND1* (ribosome biogenesis) promotes proliferation *via* CDK4/6 activation and MYC-driven oncogenic translation (Dementyeva et al., 2013; Favasuli et al., 2024); *ECT2* (RB pathway) disrupts cell cycle checkpoints through RB1 phosphorylation, exacerbating CA-induced mitotic errors (Wu et al., 2021); *ARL2* (mitochondrial translation) may induce glycolytic dependency by impairing oxidative phosphorylation, a key survival adaptation in therapy resistance (Kryukova, Kryukov & Hajek, 2016; Li et al., 2017); *KLHL12* (RNA splicing) likely modulates oncogenic RNA isoforms to enhance heterogeneity and drug resistance (Liu et al., 2023; Yu et al., 2024). Notably, *SAC3D1* integrates RNA metabolism with centrosome dynamics (Liu et al., 2020), but its MM-specific mechanisms remain unclear and are not the focus of this study.

These findings suggest that the CARG signature represents the coordinated dysregulation of CA-related pathways, consistent with established mechanisms of genomic instability in MM. Future functional validation (*e.g.*, CRISPR silencing, metabolic assays) should prioritize elucidating how perturbations in these pathways amplify CA-driven vulnerabilities, which is critical for translating this signature into targeted therapies (Ohguchi et al., 2017; Walters et al., 2018).

The six-gene CARG signature-based risk model correlates with distinct immune profiles: high-risk patients exhibit increased regulatory T cell infiltration, indicating an immunosuppressive microenvironment that favors immune escape, consistent with the signature genes’ established roles in modulating immune activity. Additionally, differential drug sensitivity (*e.g.*, CCT007093) suggests the signature’s potential to guide personalized therapy selection.

We further analyzed the association between the constructed risk model and molecular features related to centrosome amplification (CA). Given that CA status was not available in the original datasets, we used 1q21 amplification-associated genes as a surrogate indicator. 1q21 amplification is a common cytogenetic abnormality in multiple myeloma and is closely linked to chromosomal instability and centrosome dysfunction (Hanamura, 2021). Our results showed that in both the training and validation cohorts, patients in the high-risk group exhibited significantly elevated CA-related scores, and risk scores were positively correlated with CA scores. Moreover, several representative 1q21-related genes were upregulated in the high-risk group, further supporting the association between the risk model and CA-related molecular features. These findings suggest that the risk model may indirectly reflect molecular abnormalities associated with CA. It should be noted, however, that CA status was not directly assessed in this study, and the expression difference of *BCL9* in the validation cohort did not reach statistical significance, which may be attributable to cohort heterogeneity or limited sample size. Overall, by analyzing CA-related surrogate indicators, this study provides supplementary evidence supporting the link between the risk model and CA, although the underlying biological mechanisms and clinical significance require further validation through functional experiments and prospective cohort studies.

RT-qPCR validation (*n* = 5 per group) confirmed significant upregulation of *RUVBL1*, *KLHL12*, *ECT2*, *SAC3D1*, and *CCND1* in MM samples, while *ARL2* showed a non-significant upward trend. These findings align with existing mechanistic insights: *CCND1* (driving the G1/S transition) and *ECT2* (promoting CA and mitotic errors) reinforce their established oncogenic roles in MM (De Mel et al., 2014), and elevated *RUVBL1* (a MYC cofactor) correlates with advanced disease, consistent with its prognostic relevance.Meanwhile, the lack of significant difference in *ARL2* expression may be attributed to several factors. The validation sample size was small (five cases per group), which limited statistical power. Additionally, the low proportion of tumor cells in peripheral blood samples may have diluted the expression differences. More importantly, none of the datasets used in this study provided direct information on CA status, and the expression level of *ARL2* may be closely associated with CA status. If the proportion of CA-positive samples differed between the validation cohort and the transcriptomic cohorts, the overall expression trend of *ARL2* could be affected. Therefore, the expression pattern of *ARL2* in MM and its association with CA warrant further validation in larger, CA-stratified cohorts.

This study has several limitations. First, core analysis was based on bone marrow samples, with peripheral blood validation being exploratory. The inherent differences in cellular composition, low myeloma cell proportion, and high background interference in peripheral blood may have influenced the results. The small sample size and incomplete clinical data (*e.g.*, missing R-ISS score, CA status, and *ARL2*-related CA data) also restricted model evaluation and biomarker interpretation. Second, verification of causal relationships and exploration of therapeutic strategies were insufficient. The CA-independent immune cell infiltration analysis cannot exclude potential CA interference or distinguish platform-specific differences from CA-related effects, which impacts the interpretation of integrated results. Additionally, the small validation sample size and MM subtype heterogeneity may explain the non-significant trend observed for *ARL2*, while the upregulation of *KLHL12* and *SAC3D1* suggests potential roles in CA-related pathways (*e.g.*, ubiquitination), which require functional validation (*e.g.*, CRISPR knockdown) to confirm causality.

To address these limitations, future studies will: (1) expand sample sizes to include multicenter MM cohorts with complete clinical annotations, incorporate bone marrow and peripheral blood mixed samples, and optimize validation designs; (2) re-analyze *ARL2* expression in CA subgroups using GEO/TCGA datasets and explore its associations with CA and prognosis; (3) conduct cellular and animal gene knockout/overexpression experiments and prospective studies to verify target value; (4) utilize CIBERSORT or single-cell sequencing to analyze CA’s regulatory role in immune infiltration and the synergistic relationships between CA, immune characteristics, and biomarkers, enhancing prognostic factor analysis to increase study rigor and the generalizability of conclusions.

## Conclusions

This study identified six CARGs (*RUVBL1*, *ARL2*, *KLHL12*, *ECT2*, *SAC3D1*, and *CCND1*) as prognostic biomarkers for MM. These genes were successfully used to construct a risk prediction model, which demonstrated stable prognostic performance in the independent validation cohorts GSE136337 and GSE24080. The findings provide experimental evidence that the six-gene signature associated with CA is correlated with the survival of patients with MM and can be applied for risk stratification. Notably, the risk model showed correlations with MM clinical staging (ISS/RISS), immune cell infiltration patterns, TIDE scores, and sensitivity to multiple drugs (*e.g.*, CCT007093), further supporting its potential relevance in the prognostic assessment of MM.

##  Supplemental Information

10.7717/peerj.21283/supp-1 Supplemental Information 1 40 candidate genes in MM

10.7717/peerj.21283/supp-2 Supplemental Information 2 Variance inflation factor (VIF) analysis VIF analysis showed that the VIF values for risk score, age, ISS, and RISS were all ¡ 5 , indicating low multicollinearity among these independent prognostic factors.

10.7717/peerj.21283/supp-3 Supplemental Information 3 R language code

10.7717/peerj.21283/supp-4 Supplemental Information 4 PCR data processing

10.7717/peerj.21283/supp-5 Supplemental Information 5 In comparison to the normal group, the expression of CCND1 was notably higher in MM (p ¡ 0.05)

10.7717/peerj.21283/supp-6 Supplemental Information 6 In comparison to the normal group, the expression of ARL2 tended to be up-regulated in MM, the differences were not significant, potentially due to the small sample size

10.7717/peerj.21283/supp-7 Supplemental Information 7 In comparison to the normal group, the expression of RUVBL1 was notably higher in MM (p ¡ 0.05)

10.7717/peerj.21283/supp-8 Supplemental Information 8 In comparison to the normal group, the expression of ECT2 was notably higher in MM (p ¡ 0.05)

10.7717/peerj.21283/supp-9 Supplemental Information 9 In comparison to the normal group, the expression of KLHL12 was notably higher in MM (p ¡ 0.05)

10.7717/peerj.21283/supp-10 Supplemental Information 10 In comparison to the normal group, the expression of SAC3D1 was notably higher in MM (p ¡ 0.05)

10.7717/peerj.21283/supp-11 Supplemental Information 11 MIQE-checklist

10.7717/peerj.21283/supp-12 Supplemental Information 12 Analysis Flowchart The overall workflow of the study

10.7717/peerj.21283/supp-13 Supplemental Information 13 Analysis of Correlation Characteristics Related to Centrosome Amplification (A-B) Scores of centrosome amplification-related features in the training set/validation set and their relationship with risk scores (A) Training set (B) Validation set (C-D) Expression differences of representative genes in the 1q21 region in different risk groups of the training set/validation set (C) Training set (D) Validation se

## Additional Information and Declarations

### Data Availability

The following information was supplied regarding data availability: The code and data are available in the Supplemental Files.
