# Supplementary material for: Identification and validation of prognostic genes related to centrosome amplification in multiple myeloma
Source: PeerJ. 2026 Jun 4;14:e21283. doi: 10.7717/peerj.21283 (PMC13242746; doi:10.7717/peerj.21283)
Supplement: Supplemental Information 1 [file peerj-14-21283-s001.docx]

**Table S1: 40 candidate genes**

|  | symbol |
| --- | --- |
| 1 | CKAP2 |
| 2 | IL4R |
| 3 | HNMT |
| 4 | DYNC1H1 |
| 5 | CCDC85B |
| 6 | CEP164 |
| 7 | HSPA1A |
| 8 | TUBGCP3 |
| 9 | TAP1 |
| 10 | RGCC |
| 11 | GADD45A |
| 12 | TUBA1A |
| 13 | FHOD1 |
| 14 | RUVBL1 |
| 15 | DYSF |
| 16 | RAP1GAP2 |
| 17 | ARL2 |
| 18 | PROCR |
| 19 | FAM110B |
| 20 | CEP72 |
| 21 | DRD4 |
| 22 | NDC80 |
| 23 | KLHL12 |
| 24 | ECT2 |
| 25 | EFHC1 |
| 26 | PRKAR2B |
| 27 | RUVBL2 |
| 28 | TEK |
| 29 | TRAF5 |
| 30 | PLAG1 |
| 31 | AURKA |
| 32 | SAC3D1 |
| 33 | KIZ |
| 34 | MARCKS |
| 35 | RRAGD |
| 36 | ANKS1B |
| 37 | CCND1 |
| 38 | SNX10 |
| 39 | MAGI2 |
| 40 | TRAT1 |
