# Supplementary material for: Identification and validation of prognostic genes related to centrosome amplification in multiple myeloma
Source: PeerJ. 2026 Jun 4;14:e21283. doi: 10.7717/peerj.21283 (PMC13242746; doi:10.7717/peerj.21283)
Supplement: Supplemental Information 2 — VIF analysis showed that the VIF values for risk score, age, ISS, and RISS were all ¡ 5 , indicating low multicollinearity among these independent prognostic factors. [file peerj-14-21283-s002.docx]

| **Table S2 VIF values of independent prognostic factors** | | | | | | |
| --- | --- | --- | --- | --- | --- | --- |
| Variates | riskScore | Age1 | ISS11 | ISS111 | RISS11 | RISS111 |
| VIF | 1.008496 | 1.009733 | 1.574869 | 1.745276 | 1.796554 | 1.91125 |
