# Supplementary figures and images for: Identification and validation of prognostic genes related to centrosome amplification in multiple myeloma
Source: PeerJ. 2026 Jun 4;14:e21283. doi: 10.7717/peerj.21283 (PMC13242746; doi:10.7717/peerj.21283)

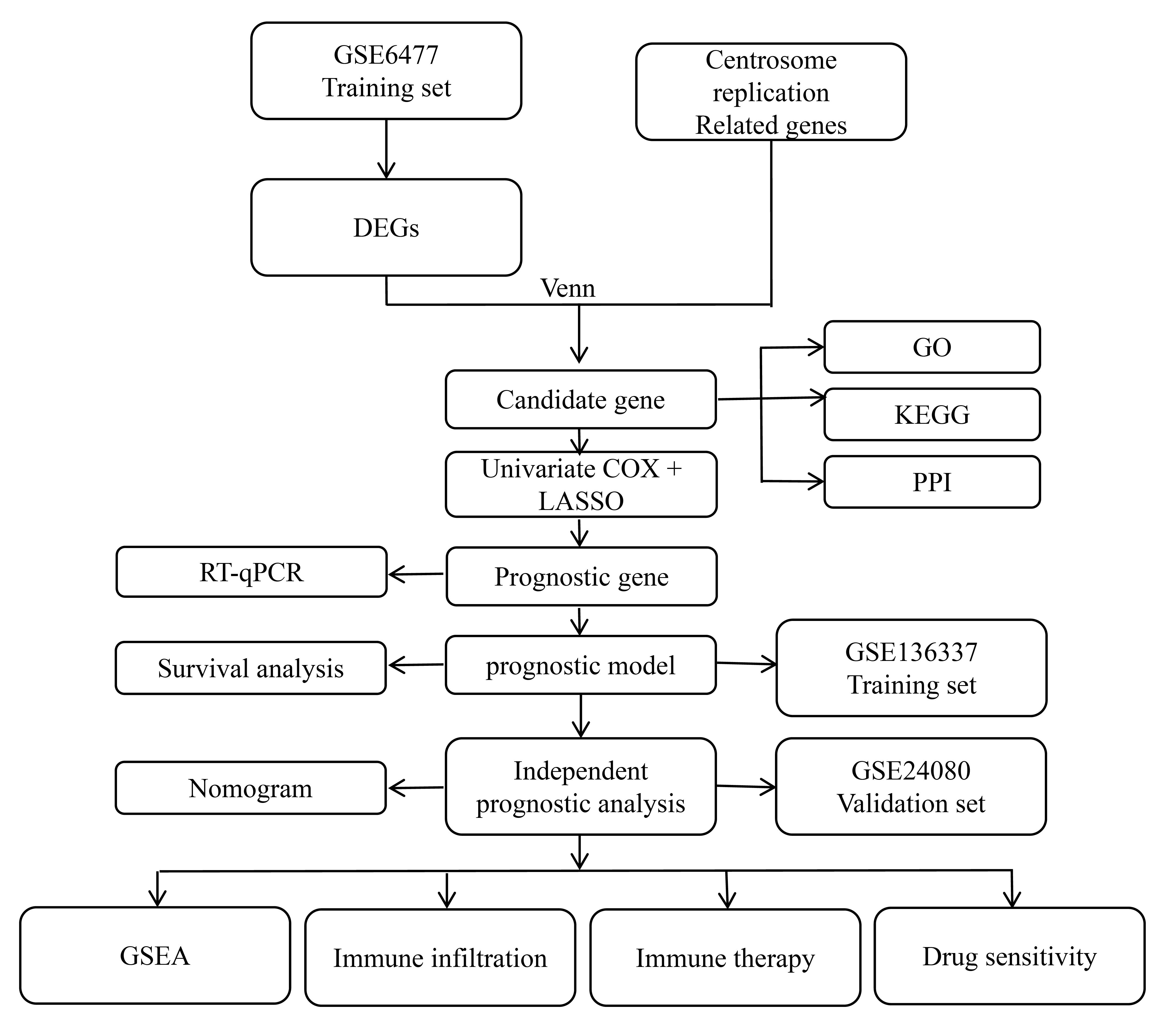

Supplement: Supplemental Information 12 — The overall workflow of the study [file peerj-14-21283-s012.tif]

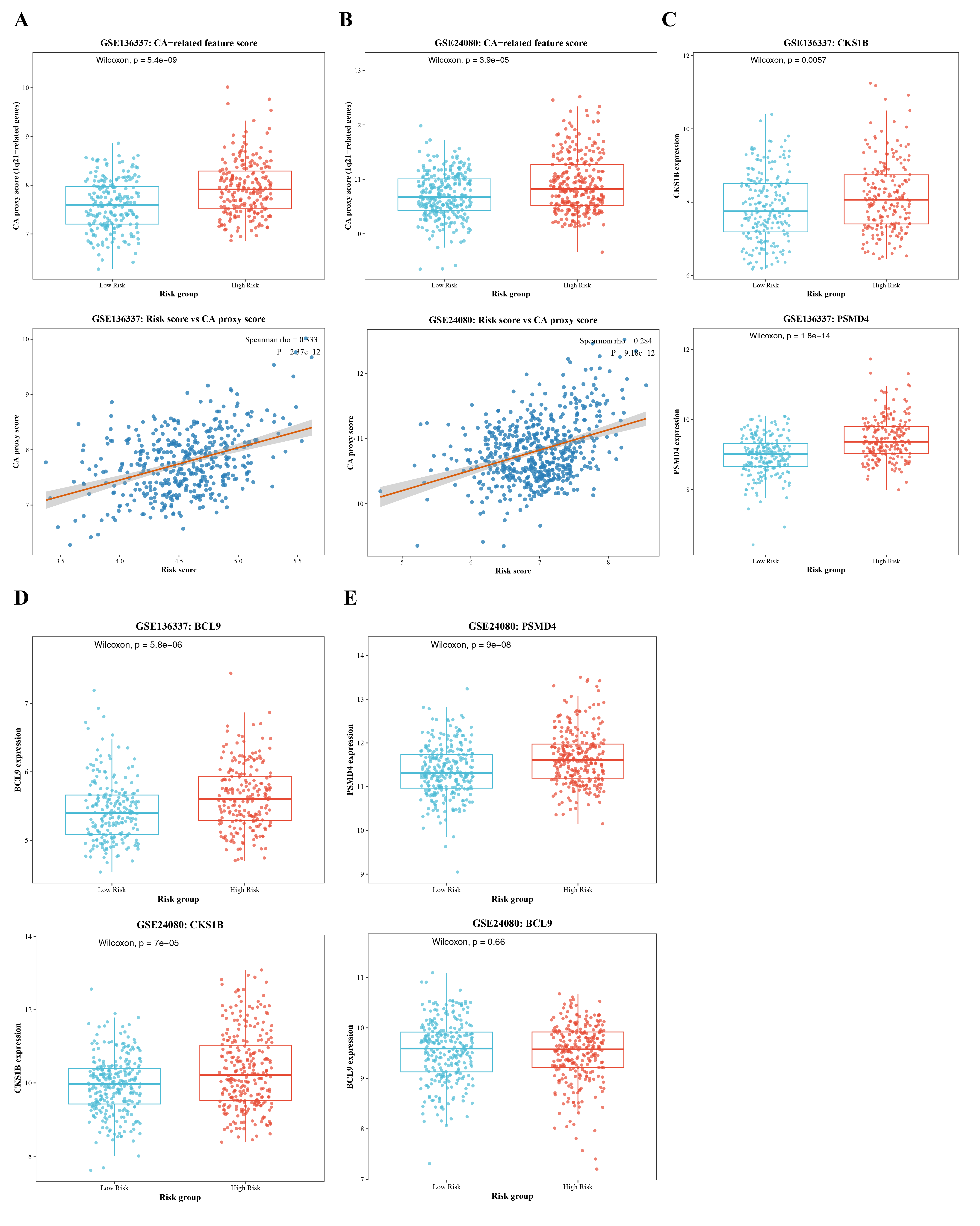

Supplement: Supplemental Information 13 — (A-B) Scores of centrosome amplification-related features in the training set/validation set and their relationship with risk scores (A) Training set (B) Validation set (C-D) Expression differences of representative genes in the 1q21 region in different risk groups of the training set/validation set (C) Training set (D) Validation se [file peerj-14-21283-s013.tif]
